# RMol: a toolset for transforming SD/Molfile structure information into R objects

**DOI:** 10.1186/1751-0473-7-12

**Published:** 2012-11-14

**Authors:** Martin Grabner, Kurt Varmuza, Matthias Dehmer

**Affiliations:** 1 Department of Biomedical Sciences and Engineering, Institute for Bioinformatics and Translational Research, University for Health Sciences, Medical Informatics and Technology (UMIT), Eduard Wallnöfer Zentrum 1, Hall in Tyrol, A-6060, Austria; 2 Laboratory for Chemometrics, Institute of Chemical Engineering, Vienna University of Technology, A-1060 Vienna, Getreidemarkt 9/166, Austria

## Abstract

**Background:**

The graph-theoretical analysis of molecular networks has a long tradition in chemoinformatics. As demonstrated frequently, a well designed format to encode chemical structures and structure-related information of organic compounds is the Molfile format. But when it comes to use modern programming languages for statistical data analysis in Bio- and Chemoinformatics, R as one of the most powerful free languages lacks tools to process Molfile data collections and import molecular network data into R.

**Results:**

We design an R object which allows a lossless information mapping of structural information from Molfiles into R objects. This provides the basis to use the RMol object as an anchor for connecting Molfile data collections with R libraries for analyzing graphs. Associated with the RMol objects, a set of R functions completes the toolset to organize, describe and manipulate the converted data sets. Further, we bypass R-typical limits for manipulating large data sets by storing R objects in bz-compressed serialized files instead of employing RData files.

**Conclusions:**

By design, RMol is a R toolset without dependencies to other libraries or programming languages. It is useful to integrate into pipelines for serialized batch analysis by using network data and, therefore, helps to process sdf-data sets in R efficiently. It is freely available under the BSD licence. The script source can be downloaded from http://sourceforge.net/p/rmol-toolset.

## Background

To solve many tasks in Bio- and Chemoinformatics, the analysis of chemical and biological structures represented by networks has been proven powerful [1,2]. A typical problem in this area is to characterize the structure of molecular networks quantitatively by using graph measures [3-7] or to predict physicochemical properties of the molecules by taking structural features into account [8].

For quantifying structural information of molecular networks, one often needs quantitative or comparative network measures to analyze the structure of the underlying networks [1,2]. For instance, Dragon[9] is a commercial and well-known software to calculate so-called molecular descriptors from SD/Molfile data [10] and other data formats specializing in chemical structures. But when using the programming environment R[11], there is yet no interface to employ structural information of molecular networks encoded by SD/Molfile data.

To tackle this problem and, hence, to spread out the usage of R in chemically and biologically-driven disciplines, we develop an R toolset for transforming SD/Molfile structure information into R objects. As structural information of the networks is now available in R, we hope that our tool may stimulate the Bioinformatics community to explore problems centered around chemical and molecular networks by using existing R packages.

## Tools for graph analysis

In this section, we briefly sketch some tools for analyzing graphs by using R. An extensive review of such tools can be found in [12,13].

Among other environments suitable for graph analysis [14], the script language R has gained much importance. Not only because basic functions are allocated by R packages such as the packages graph[15] from Bioconductor[16] and igraph[17] but also because of packages such as QuACN[18]. The latter contributed extensively to analyze networks quantitatively [18] with R. Note that QuACN is an R-tool for calculating ca. 150 quantitative network measures which can be mostly interpreted as complexity indices [19].

Also, Guha [20] developed a set of wrapper functions providing R user access to the functions and objects of the CDK [21] representing a Java framework for cheminformatics. The chemoinformatics package ChemMineR[22] written in R includes in updated versions functions which are capable of reading and extracting structural information from different data formats including SD-files. But we emphasize that both packages are conceptionally focused on the inspection of single networks or comparison of limited data sets, in contrast to the RMol script collection which fits well into workflows with serialized pipelines. To reduce dependencies, we extract SD-file information with our own R parser. Also, to avoid repetitive transformation processes we store the chemical and molecular network information in R objects.

**Figure 1:**
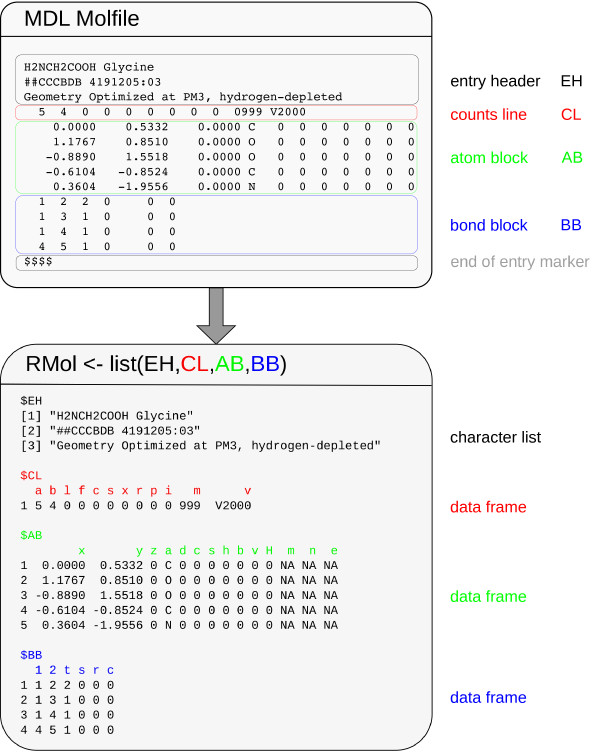
**Molfile <—> RMol mapping.** Four structural elements (entry header EH, counts line CL, atom block AB, bond block BB) from a Molfile are transformed to list elements of an RMol object. Column labels of the RMol data frames CL, AB, BB are named according to the CT (Chemical Table) file specifications [23].

In this report, we present an R toolset for linking available R functions with existing data collections representing chemical structures by mapping structural information of MDL-Molfiles to an R object called RMol. Figure 1 illustrates the lossless mapping from Molfile information to a list consisting of four elements that represents an RMol object.

## Results and discussion

Besides the definition of the novel RMol object for encoding chemical structure information by using R, we develop a set of functions for the programming environment R to accomplish and facilitate efficient graph-based analysis of the underlying molecular networks, e.g., chemical structures. More precisely, this tool covers the following functionalities: 

• Importing chemical structure data from an SD-file (Molfile format) into R.

• Handling of RMol data sets as serialized bz-compressed files (to bypass memory limits).

• Providing simple statistics of chemical structures or structure data sets in RMol format.

• A filter for selecting chemical structures and reorganizing data collections in RMol format.

• Generating adjacency matrices or connection tables from chemical structures in RMol format.

• Converting RMol objects into attribute-extended graphNEL objects. By doing so, this links directly to R packages for graph analysis (e.g., see graph, igraph, QuACN).

In the following, we explain some items of RMol in more detail. The function Sdf2RMol has been developed to process SD-files and convert chemical structure information from the Molfile portions into RMol objects.

Concretely Sdf2RMol represents a working script, which combines an entry picking routine (pickSdfEntry) with an RMol specific parser (parseSdfEntry) using regular expressions to scan the Molfile sections of SD-files according to the CT-file format specifications [23]. Moreover Sdf2RMol completes the conversion pipeline with error logging and internal routines for checking feeded entries for consistency and plausibility. Finally the resulting R objects are streamed as data sets into serialized bz-compressed files.

These files are denoted with the file ending .Rbz and referred to as ”Rbz-files”. Rbz-files help to bypass R-typical memory limits for huge data collections and are useful storage containers for any R object. By design, Rbz-files contain the R objects as serialized list elements *S*[*i*], where S[i]=list(objectname[i],objectcontent[i]). They are a useful data source for any R driven process pipeline.

The functions RData2Rbz and Rbz2RData allow the transformation of the serial Rbz-format to standard RData-format and vice versa. For users who are not familiar with connection manipulation in R, we also include the functions RbzOpen, NextRbzObject, RbzClose to alleviate the handling of Rbz-files.

To extract and summarize properties of Rbz-packed RMol data sets, the functions RbzSummary and RbzSummaryReport are useful. To manipulate and split these data sets RbzFilter is available.

The raw graphNEL class, as defined in the R package graph is sufficient to build representations for graphs without vertex and edge labels. However, to perform the analysis of labeled graphs (e.g., graphs representing chemical structures with hetero atoms and bond types) by using the QuACN package, graphNEL needs to be extended with bond and atom attributes. RMol contains the function RMol2QuACNgN to pack the relevant information into these attribute-extended graphNEL objects. All RMol functions are put together in one R script. After sourcing this script all functions will be available to support in preparing chemical structure data for analyzing molecular networks.

## Conclusions

In this report, we presented an R toolset to convert the structure information of molecular graphs encoded by SD/Molfiles into R objects. It complements existing packages capable of reading SD-file information by easying batch processing and using pure R scripts without dependencies. In combination with R packages designed for analysing graph and network properties it represents a connector module for R workflows which process structure information from SD-files. This toolset can also support other R packages for analyzing networks structurally and, thus, makes a further contribution towards demonstrating the power of R for network analysis in Chemo- and Bioinformatics.

So far, it was not common to investigate SD-file data collections by using R and packages thereof. The new toolset RMol may encourage the community to spread out the usage of R for chemically and biologically driven areas.

## Competing interests

The authors declare that they have no competing interests.

## Authors’ contributions

MG developed the R package. KV also participated in this process. MD, MG and KV wrote the manuscript. All authors read and approved the final manuscript.
